# α_1_-Acid Glycoprotein and Dietary Intake in End-Stage Renal Disease Patients

**DOI:** 10.3390/nu13113671

**Published:** 2021-10-20

**Authors:** Małgorzata Maraj, Paulina Hetwer, Beata Kuśnierz-Cabala, Barbara Maziarz, Paulina Dumnicka, Marek Kuźniewski, Piotr Ceranowicz

**Affiliations:** 1 Department of Physiology, Faculty of Medicine, Jagiellonian University Medical College, Grzegórzecka 16 St., 31-531 Kraków, Poland; malgorzata.maraj@uj.edu.pl (M.M.); piotr.ceranowicz@uj.edu.pl (P.C.); 2 Faculty of Medicine, Dietetics, Jagiellonian University Medical College, Anny 12 St., 31-008 Kraków, Poland; paulina.hetwer@op.pl; 3 Chair of Clinical Biochemistry, Department of Diagnostics, Faculty of Medicine, Jagiellonian University Medical College, Skawińska 8 St., 31-066 Kraków, Poland; barbara.maziarz@uj.edu.pl; 4 Department of Medical Diagnostics, Faculty of Pharmacy, Jagiellonian University Medical College, Medyczna 9 St., 30-688 Kraków, Poland; paulina.dumnicka@uj.edu.pl; 5 Chair and Department of Nephrology, Faculty of Medicine, Jagiellonian University Medical College, Jakubowskiego 2 St., 30-688 Kraków, Poland; marek.kuzniewski@uj.edu.pl

**Keywords:** hemodialysis, end-stage kidney disease, malnutrition, dietary intake, α_1_-acid glycoprotein, appetite

## Abstract

Management of end-stage renal disease (ESRD) patients requires monitoring each of the components of malnutrition–inflammation–atherosclerosis (MIA) syndrome. Restrictive diet can negatively affect nutritional status and inflammation. An acute-phase protein—α_1_-acid glycoprotein (AGP), has been associated with energy metabolism in animal and human studies. The aim of our study was to look for a relationship between serum AGP concentrations, laboratory parameters, and nutrient intake in ESRD patients. The study included 59 patients treated with maintenance hemodialysis. A 24 h recall assessed dietary intake during four non-consecutive days—two days in the post-summer period, and two post-winter. Selected laboratory tests were performed: complete blood count, serum iron, total iron biding capacity (TIBC) and unsaturated iron biding capacity (UIBC), vitamin D, AGP, C-reactive protein (CRP), albumin, prealbumin, and phosphate–calcium metabolism markers (intact parathyroid hormone, calcium, phosphate). Recorded dietary intake was highly deficient. A majority of patients did not meet recommended daily requirements for energy, protein, fiber, iron, magnesium, folate, and vitamin D. AGP correlated positively with CRP (R = 0.66), platelets (R = 0.29), and negatively with iron (R = −0.27) and TIBC (R = −0.30). AGP correlated negatively with the dietary intake of plant protein (R = −0.40), potassium (R = −0.27), copper (R = −0.30), vitamin B_6_ (R = −0.27), and folates (R = −0.27), p < 0.05. However, in multiple regression adjusted for confounders, only CRP was significantly associated with AGP. Our results indicate that in hemodialyzed patients, serum AGP is weakly associated with dietary intake of several nutrients, including plant protein.

## 1. Introduction

Excess body fat appears to be a strong risk factor for the development and progression of kidney disease [1]. However, once chronic kidney disease (CKD) is acquired, obesity may be associated with favorable outcomes especially among the group of patients receiving hemodialysis (HD) [2,3,4,5,6] as their body mass index (BMI) and nutrient intake decrease with the dialysis time [7]. Stenvinkel et al. distinguish two types of malnutrition [8]. One is easily reversed by nutritional intervention, the other is characterized by oxidative stress, inflammation, and increased catabolic rate, which can easily progress into cachexia and muscle wasting. Pro-inflammatory mediators induce hepatic synthesis of positive acute-phase proteins (e.g., C-reactive protein—CRP and α_1_-acid glycoprotein—AGP) and inhibit the hepatic generation of negative acute-phase reactants, such as albumin and prealbumin [9]. Released cytokines, including interleukin (IL)-6, contribute to catabolism of skeletal muscle proteins and interfere with the satiety center and delay gastric emptying [9]. The coincidence of inadequate elimination of nitrogenous waste products, uremic toxicity, acidosis and increased serum concentrations of the inflammatory markers such as CRP, IL-6, and tumor necrosis factor α (TNF-α) all contribute to the loss of appetite in the group of HD patients [10,11].

AGP (orosomucoid–ORM) is an acute-phase protein which aims at protecting the host against the detrimental side effects of an excessive inflammatory reaction—it can inhibit proliferation of lymphocytes, chemotaxis of neutrophils and superoxide production as well as prevent aggregation of platelets [12]. It is mainly produced in the liver—glucocorticoids, TNF-α, IL-1, IL-8, IL-11, and IL-6 have been reported as major regulatory mediators of *ORM* gene expression in hepatocytes [13]. However, in obese mice, AGP is also secreted from adipocytes in response to metabolic and inflammatory signals [13]. Nutritional status could affect *ORM* expression in metabolic organs such as the liver and fat [14]. In human, increased level of AGP correlated with BMI, body fat mass, serum leptin, and fasting plasma glucose level [13]. It has been hypothesized that the adipose tissue preferentially recognizes and responds to metabolic changes, while the liver AGP production is more sensitive to acute inflammation. A study on mice also revealed that AGP could function as an agonist for leptin receptor—it activates the Janus kinase (JAK) 2–signal transducer and activator of transcription STAT 3 pathway in the hypothalamus [14]. According to Agra et al., downregulating of leptin by high AGP could be a protective mechanism in regulating food intake and energy homeostasis [15]. AGP may integrate inflammatory and metabolic signals to modulate immune responses to protect adipose tissue from excessive inflammation and thereby from metabolic dysfunction [16].

Higher BMI, with an emphasis on lean body and muscle mass [17] are protective factors in malnourished patients and the weight gain and simultaneous downplay of the inflammatory response could add years to their survival. Restrictive diet and inadequate nutrient intake negatively affect nutritional status of HD patients. AGP production may be related to energy metabolism and adipose tissue metabolism and could be a marker of nutritional status. The aim of this study was to evaluate the nutritional intake of ESRD patients treated with maintenance HD and to look for a long-term relationship between serum AGP concentrations and nutrient intake, as well as results of laboratory tests used to monitor patients’ state.

## 2. Material and Methods

### 2.1. Study Participants

The prospective study included 59 adult patients in ESRD undergoing maintenance HD (three times a week), who were recruited from the Department of Nephrology of Jagiellonian University Medical College in Krakow, Poland [18]. We included patients with stable course of the disease during at least two months preceding the onset of the study. Patients with infections or acute inflammatory conditions at the time of assessment were excluded. The patients participated voluntarily and a written informed consent was granted by all subjects. The study protocol received the approval of the Bioethics Committee (number 1072.6120.105.2018 issued on 20 April 2018).

### 2.2. Clinical and Nutritional Assessment

At the onset of the study, data on comorbidities, duration of treatment, height, and body mass were obtained from hospital documentation, and a self-constructed questionnaire was distributed and completed by patients on their own or with author’s assistance (P.H.). The self-contained questionnaire asked about selected lifestyle aspects, including physical activity, diet, and supplementation, and was described in detail in Maraj et al. [18]. A 24 h dietary recall assessment was conducted twice: in November 2018 and February/March 2019. Each assessment collected information on dietary intake during one day of the week and one day of the weekend.

### 2.3. Laboratory Tests and Calculations

The study analyzed the results of two separate laboratory examinations performed in November 2018 and February/March 2019, in concordance with dietary recall. Patients’ blood samples were collected routinely for periodic examination performed to monitor the patients’ health state. The blood for laboratory tests was collected before the mid-week hemodialysis session. The blood was collected by puncturing the vein of the forearm to Sarstedt Company’s closed systems according to evidence-based laboratory medicine.

For biochemical and immunochemical tests, serum was used. Serum iron, total iron-binding capacity (TIBC), unsaturated iron-binding capacity (UIBC), total and ionized calcium, phosphates, total vitamin D (25OH-D_2_ and -D_3_), intact parathyroid hormone (iPTH), albumin concentration, and concentrations of fms-like tyrosine kinase 1 (sFlt-1) were measured on a Cobas 8000 analyzer (Roche Diagnostics, Mannheim, Germany). Acute-phase proteins prealbumin, AGP, and CRP in sera were measured using an immunonephelometric method on a Nephelometer II analyzer (BN II) (Siemens Healthcare, Erlangen, Germany). Complete blood count was conducted in K_2_EDTA blood using a Sysmex XN hematology analyzer (Sysmex Corporation, Kobe, Japan). Laboratory tests were carried out in the Diagnostic Department of University Hospital in Krakow, Poland.

Dialysis adequacy was assessed using Kt/V, calculated with the formula of Daugirdas [19]: Kt/V = −ln (R − 0.008t) + (4 − 3.5 × R) × U/W, where R is the ratio of post- to pre-dialysis serum urea concentrations, t denotes the time of dialysis session in hours, U denotes the ultrafiltration volume during the HD session (in L), and W denotes the post-dialysis body weight in kg. The normalized protein catabolic rate (nPCR) was estimated based on the simplified equation of Garred et al. [20]: nPCR = 0.01575 × (1 + 0.0569 × Kt/V) × Kt/V × C_0_ × (1 − R)/(−ln R) + 0.17, where C_0_ is the pre-dialysis blood urea nitrogen in mg/dL and R denotes post- to pre-dialysis blood urea nitrogen. Both Kt/V and PCR were assessed twice, in November 2018 and February/March 2019, and the arithmetic means of the two values were reported.

### 2.4. Statistical Analysis

Dietary intake may vary between different seasons throughout the year, because of changing availability of products as well as circannual rhythms leading to fluctuations in the release of feeding hormones, which may affect appetite [21,22]. To account for this seasonality, we calculated arithmetic means from the collected data on dietary intake and the available results of laboratory tests obtained at the two time points specified above (post-summer, November 2018, and post-winter, February/March 2019). We report the results based on the arithmetic mean values. For the quantitative variables, mean ± standard deviation (SD) and median (lower–upper quartile) were shown depending on the normality of the distribution. The normality was assessed using the Shapiro–Wilk test. The Spearman rank coefficient was used to study simple correlations. Multiple linear regression was used to evaluate which variables were independently associated with mean AGP concentrations: the unadjusted model included the variables significantly correlated with AGP (*p* < 0.05) as the independent variables; the adjusted model included the confounders such as age, sex, dialysis vintage, dialysis dose, and comorbidities, as specified in Results. The right-skewed variables were log-transformed before being included in the linear regression model. Results were considered significant at *p* < 0.05. The statistical analysis was conducted using Statistica 12.0 (StatSoft, Tulsa, OK, USA) software.

## 3. Results

The study included 59 patients with end-stage renal failure treated with maintenance HD. The average age of participants at the start of the study was 58 years and the duration of renal replacement therapy ranged from 3 to 392 months (Table 1). Among patients, 4 (6.8%) were underweight (BMI < 18.5 kg/m^2^), 26 (44.1%) had normal BMI, 16 (2.1%) were overweight (BMI in the range 25.0–29.9 kg/m^2^), and 13 (22.0%) were obese (BMI ≥ 30 kg/m^2^). Diabetes was diagnosed in 36% of the studied group while cardiovascular comorbidities were present in 86% (Table 1). There were 12 patients who had undergone renal transplantation in the past and returned to HD treatment following the transplant failure (Table 1). Kt/V was above 1.2 in 48 (81.4%) patients and nPCR was above 1 g/kg/day in 35 (59.3%) patients.

The mean results of laboratory tests (i.e., arithmetic mean of the results obtained during the post-summer and post-winter assessment, as specified in Methods) revealed the abnormalities characteristic for ESRD, including low RBC, hemoglobin and hematocrit, low serum concentrations of vitamin D, and low serum albumin in about 25% of patients. Increased serum concentrations of acute-phase proteins were observed in a substantial proportion of the studied group: CRP above 5 mg/L was recorded in 47% of patients and AGP above the reference range in 42% of patients (Table 2). Serum iron and UIBC were within the reference intervals in most patients, while TIBC was decreased (Table 2).

Dietary intake based on mean values recorded at the two time periods was highly deficient (Table 3). The lower recommended dietary allowance (RDA) threshold for energy was met by only 17% of patients. Protein RDA was reached by 10% of the studied patients. A total of 51 (86%) patients consumed less than 1 g/kg of protein (within the group mean protein intake was 0.7 g/kg). The reported dietary protein intake was significantly lower as compared to nPCR multiplied per dry body weight (52.5 ± 14.9 vs. 79.4 ± 25.7 g/day; *p* < 0.001). None of the patients consumed the required amount of magnesium, or folate, and only 2% met the daily requirements for fiber and vitamin D. A total of 12%of patients provided adequate amount of zinc and vitamin E in their diets. Fewer males consumed sufficient amounts of iron as well as zinc as compared to females. The B-vitamins intake (B_1_, B_2_, B_3_, B_6_, B_12_) was highly deficient among over 60% of patients.

**Figure 1:**
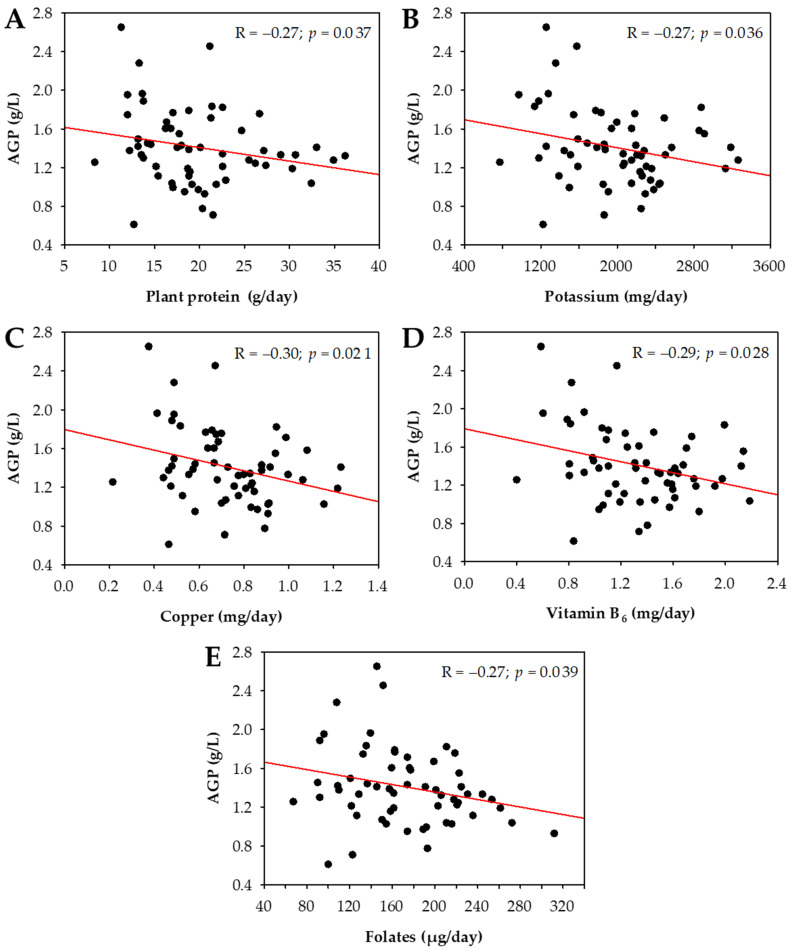
Correlations between serum concentrations of AGP and the intake of selected nutrients based on 24 h recall: plant protein (**A**), potassium (**B**), copper (**C**), vitamin B_6_ (**D**), and folates (**E**). Data are presented as arithmetic means of values obtained in post-summer and post-winter assessment.

Mean serum AGP concentrations did not differ between men and women (*p* = 0.3) and did not correlate with age (R = 0.14; *p* = 0.3), HD vintage (R = 0.08; *p* = 0.5), Kt/V (R = −0.01; *p* > 0.9), or BMI (R = 0.11; *p* = 0.4). No differences in serum AGP were observed between patients with or without diabetes (*p* = 0.1), with or without cardiovascular comorbidities (*p* = 0.4), or between patients in distinct BMI categories (*p* = 0.9). Moreover, we observed no association between serum AGP and nPCR (R = −0.01; *p* = 0.9). Serum AGP correlated positively with serum CRP concentrations, platelet counts, and sFlt-1 concentrations, and negatively with iron and TIBC (Table 4). Moreover, AGP correlated negatively with dietary intake of plant protein, potassium, copper, vitamin B_6_, and folates (Figure 1). We did not observe significant associations between serum AGP and animal (R = −0.15; *p* = 0.3) or total (R = −0.19; *p* = 0.1) protein intake.

In multiple linear regression, log-transformed serum concentration of CRP and log-transformed platelet count were significantly and independently associated with log-transformed serum AGP concentration (Table 5). The dietary intakes of all the nutrients that correlated with serum AGP in simple analysis were significantly interrelated (R > 0.70). Therefore, we only included the intake of plant protein in the model presented in Table 5. We further assessed whether any of the dietary intakes was associated with log (serum AGP) independently of log (CRP), but the analysis revealed no significant associations.

## 4. Discussion

Our results indicate that in ESRD patients treated with maintenance HD, serum AGP is weakly associated with dietary nutritional intake. The increased serum AGP concentrations reflect chronic inflammation and are most significantly associated with serum CRP. Nonetheless, the dietary assessment of HD patients revealed highly deficient diet and our results do not exclude the possibility that a more adequate diet may reduce the chronic inflammation in this group of patients.

In our study, 86% of patients had caloric food intake below the recommended range, whereas 83% did not meet the lower threshold for energy (25 kcal/kg). None of the patients met the RDA for magnesium or folate. Less than 10% of patients consumed sufficient amounts of vitamin B_1_ and iron. Less than 50% had adequate intake of vitamins B_3_, B_6_, B_12_, and E. Vitamin D intake with the diet was similarly deficient, however, 25% of patients followed vitamin D supplementation.

HD patients are often advised to limit their potassium and phosphorus intake due to the risk of hyperkalemia and hyperphosphatemia, however, many beneficial nutrients are concomitantly eliminated from their diets following reduced consumption of fruit and vegetables (regarded as the main source of potassium) and protein-rich food (often associated with high phosphorus levels) [25]. Low intake of fruit and vegetables in the studied group of patients is reflected by the fact that the fiber RDA value was reached only by 2% of patients, and similarly the total protein intake was highly deficient. It has been shown that the differentiation between animal and plant protein could compensate for this deficiency. Since humans do not express the enzyme phytase, the bioavailability of phosphorus derived from plants is lower as phytic acid or phytate comprise a major portion of total phosphorus [26]. Urinary excretion of phosphorus (a surrogate marker of dietary phosphate absorption) was found to be higher in people on meat-based diet compared to those on plant-based, and a vegetarian diet after one week significantly decreased serum phosphorus levels [27].

The components of a balanced diet play a part in the regulation of the immune system and inflammation. In light of frequently occurring MIA syndrome, diet may be of critical importance for maintaining HD patients’ quality of life. The dialysis procedure itself may promote wasting by removing such nutrients as amino acids, peptides, protein, glucose, water-soluble vitamins, and other bioactive compounds [28], whereas increased oxidative stress can impair nutrient absorption and their transport to target tissues [29], causing deficiency of several critical trace elements and vitamins [29,30,31].

The most recent Kidney Dialysis Outcome Quality Initiative (KDOQI) guidelines [23] state based on expert opinion that HD patients should be encouraged to eat a diet which provides adequate intake of all vitamins and minerals to meet nutrient needs i.e. in the amount established as RDA. However, it is also advised to regularly assess dietary vitamin intake and in case of patients with inadequate intake of water-soluble vitamins and/or essential trace elements supplementation to treat micronutrient deficiencies can be considered [23]. Nutrition guidelines suggest prescribing folate, vitamin B_12_, and/or B-complex supplement based on clinical signs and symptoms [23]. Since there is little evidence that routine supplementation of selenium or zinc improves nutritional, inflammatory, or micronutrient status, their supplementation is discouraged [23]. Similarly, vitamins A or E should not be routinely supplemented, however, in cases when their supplementation is justified, high doses should be avoided and patients monitored for potential vitamin toxicity. In HD adults who are at risk of vitamin C deficiency, it is advised to consider supplementation to meet the recommended intake of at least 90 mg/day for men and 75 mg/day for women [23]—the RDA values which in our study were met by only 17% of men and 26% of women.

Due to multiple external factors which impact malnutrition in HD patients, appetite control seems to be one of the crucial elements which could improve the nutritional status of patients. In order to prevent malnutrition in HD patients, all contributors to anorexia cachexia should be downregulated and patients at risk of developing malnutrition should be screened for and identified using different dietary assessment techniques.

Among methods used for screening insufficient nutrient intake are dietary interviews, food frequency questionnaires, and 3 day food recalls, which may be considered as alternative methods of assessing dietary energy and protein intake [32]. The main advantage of dietary interviews over other assessment methods is that they also involve an element of qualitative assessment, whereas in order to effectively plan nutrition interventions it is important to assess factors beyond dietary intake, e.g., medication use, knowledge, beliefs, attitudes, behavior and access to food, depression, cognitive function, etc. [32], as recommendations aligned with patient values and preferences may be more easily accepted, implemented, and adhered to [33].

In daily clinical practice, however, dietary interviews may be regarded as time consuming and difficult to perform, therefore a seven-point subjective global assessment (SGA) has been proposed as another valid and reliable tool for screening malnourished patients. It takes into account a patient’s weight change, dietary intake change, gastrointestinal symptoms, functional capacity and physical examination (loss of subcutaneous fat, muscle wasting, fluid retention) as reported by a patient or evaluated by a clinician. In adults on maintenance HD, the Malnutrition Inflammation Score may be used to assess nutritional status, which, apart from the seven components included in the SGA, additionally takes into account components such as body mass index, serum albumin level, and total iron-binding capacity [34].

Laboratory assessment markers such as normalized protein catabolic rate (nPCR), serum albumin, and/or serum prealbumin can also be a valid, quick, and useful method for estimating nutritional intake of patients. However, it must be noted that they should not be interpreted in isolation as they are influenced by non-nutritional factors such as inflammation, liver failure, volume expansion, and dialysate or urinary protein losses [32].

PCR was found to reflect dietary protein intake in steady state patients i.e. not overly catabolic (e.g., in consequence of inflammation) nor anabolic (with an increased build-up of protein e.g., in the process of recovery after infection). At the same time, PCR has been shown to overestimate protein intake when daily protein intake was below 1 g/kg and underestimate it when the daily protein intake was above 1 g/kg [23]. In our study nPCR was lower when compared with the dietary recall results.

Similarly, the specificity of albumin is limited as its levels may be lower in patients with inflammation or under stress and higher in patients following recovery [23]. In adults with ESRD on maintenance dialysis, lower serum albumin level may be regarded as a more explicit predictor of hospitalization and mortality [32]. As for prealbumin, another widely used serum marker of nutritional status characterized by a much shorter half lifetime than that of albumin, its specificity is also limited when infection or inflammation are present [23]. In our study we observed the serum albumin below the reference range among 17% of patients, whereas prealbumin was below the lower reference limit in 8% of patients.

In light of MIA syndrome frequently developed in HD patients, positive acute-phase proteins such as CRP, AGP, ferritin, and ceruloplasmin could be used to identify the presence of inflammation in individuals with low serum albumin or prealbumin levels and possibly for predicting outcome. The KDOQI Clinical Practice Guidelines for Nutrition in Chronic Renal Failure suggest that AGP may be more specific than CRP for detecting inflammation in MD patients [32]. In our previous study [18], we calculated a prognostic inflammatory and nutritional index (PINI), a clinical assessment tool which aggregates serum CRP, AGP, prealbumin, and albumin concentrations into a single score [35], cancer serum index (CSI) [36], and prealbumin to CRP ratio to account for the multifactorial complexity of malnutrition in HD patients.

A major aspect contributing to malnutrition and cachexia in HD patients is reduced appetite. In the Dialysis Outcomes and Practice Patent Study (DOPPS), the risk of death was 57% higher for patients who reported being extremely bothered by a lack of appetite during the preceding four weeks compared with those who did not [37]. Uremic toxicity, acidosis, and the accumulation of nitrogen-containing products from dietary and intrinsic protein catabolism may distort taste, smell, and blunt appetite of ESRD patients [38,39]. High levels of urea and dimethyl and trimethyl amines, and low levels of zinc, might be associated with decreased taste perception [40], while some patients may complain of metallic taste in the mouth leading to selectivity in the chosen products and subjective aversion to food—in the study conducted by Dobell et al., red meat was among the most unpopular foods [41].

The mechanism of the loss of appetite in HD patients is complex, multifactorial and can be explained via different mechanisms [42]. Some patients suffer from gastrointestinal changes and hormonal imbalance which aggravate the symptoms and aversion to foods. Serotonin has been considered the final and key element in appetite suppression [43], whereas in uremic patients, high plasma and brain levels of tryptophan, the precursor of serotonin, have been observed [10]. The lack of appetite can also be triggered by circulating cytokines, which interfere with and affect the satiety center in the hypothalamus [9,44,45,46]. In the study by Carrero et al. poor appetite was associated with increased inflammation (higher serum concentrations of IL-6 and CRP), and a worse nutritional status as well as worse clinical outcome [45]. There is evidence that cytokines and their corresponding receptors are present in the neuroendocrine system and brain [20]. In laboratory animal species, IL-1, IL-6, and TNF-α have been found to modulate intermediary metabolism of carbohydrate, fat, and protein substrates, regulate hypothalamic-pituitary outflow, and act in the brain to reduce food intake [47]. Proinflammatory cytokines may alter appetite and metabolic rate by acting on the central nervous system by modifying the release and/or function of several key neurotransmitters [10,11]. Recent data have shown that lipopolysaccharides, TNF-α, and IL-1 present in uremia stimulate leptin expression by signaling through the central melanocortin system [48,49,50]. Also, some experimental studies suggest that AGP may be involved in regulating satiety and anorexigenic effect and that its elevated concentration could induce appetite loss via the leptin receptor mechanism [14,51]. Leptin is a hormone most directly associated with food intake—it is released by adipocytes and is responsible for the feeling of satiety. It has been observed that leptin is significantly elevated in dialysis patients, even after correction for BMI [44].

In our study, higher intake of several nutrients was weakly associated with lower AGP level. However, after adjustment for multiple confounders, serum AGP was only associated with serum CRP, reflecting the chronic inflammation observed in HD patients. The chronic inflammation is interrelated with nutritional status, the deranged appetite control being one of the links. Based on our results, we may hypothesize that a less restrictive diet may contribute to reduced inflammation in ESRD patients treated with HD, however, this hypothesis must be verified in larger prospective trials.

## 5. Limitations

Our results were based on correlations, which were not equivalent to direct relationships between AGP and the selected nutrients. We were not able to confirm the associations between serum AGP and diet in multiple regression, which may indicate indirect relationships or may be associated with a limited number of studied patients. Data on dietary intake of nutrients were obtained via 24 h recall, which is prone to distortions [52,53]. However, in the studied group of patients we were not able to measure the concentration of each nutrient by means of adequate laboratory tests.

## 6. Conclusions

Adequate intake of nutrients may counteract increased catabolic processes and attenuate inflammation in HD patients. However, HD patients often do not consume enough nutrients and frequently suffer from the lack of appetite. In our study, higher intake of plant protein, potassium, copper, vitamin B_6_, and folates in ESRD patients was associated with lower AGP level.

The KDOQI guidelines [32] recommend the control of nutritional status of patients in order to predict morbidity/mortality, but they also emphasize that there is no single measurement that provides a comprehensive indication of protein–energy nutritional status. Malnutrition may be identified with greater sensitivity and specificity using a combination of factors. However, our results do not support incorporating AGP measurements into the standard assessment of HD patients.

## Figures and Tables

**Table 1:** Clinical characteristics and laboratory parameters of the study group.

Characteristics	HD Patients (n = 59)
Age, years	57.9 ± 14.1
Female, n (%)	24 (40.7)
BMI, kg/m^2^	25.1 ± 5.3
Dialysis duration time, months	67.5 (23–159.5)
Treatment with phosphate binders, n (%)	46 (77.9)
Vitamin D supplementation, n (%)	25 (42.4)
Treatment with erythropoietin analogues, n (%)	28 (47.5)
Iron supplementation, n (%)	13 (22.0)
History of kidney transplantation, n (%)	12 (20.3)
Comorbidities:	
Diabetes, n (%)	21 (35.6)
Cardiovascular disease, n (%)	51 (86.4)
Dyslipidemia, n (%)	20 (33.9)
Hypertension, n (%)	49 (83.1)
Physical activity level:	
Low, n (%)	47 (79.7)
Moderate, n (%)	12 (20.3)
Kt/V	1.43 (1.22; 1.62)
nPCR, g/kg/day	1.11 ± 0.32

BMI—body mass index, ESRD—end-stage renal disease, nPCR—normalized protein catabolic rate.

**Table 2:** The results of selected laboratory tests in the studied group of ESRD patients. Data are shown as arithmetic means of the results obtained in post-summer and post-winter assessment.

Laboratory Parameters	HD Patients (n = 59)	Reference Interval
RBC (×10^6^/μL)	W: 3.58 ± 0.39; M: 3.63 (3.44; 3.86)	W: 3.5–5.0; M: 4.5–6.5
HGB (g/dL)	W: 11.0 ± 0.9; M: 10.9 ± 1.3	W: 11.0–15.0; M: 12.0–17.0
HCT (%)	W: 34.1 ± 3.2; M: 33.5 ± 3.8	W: 37.0–47.0; M: 40.0–54.0
MCV (fL)	91.9 (88.8–95.8)	82.0–92.0
MCHC (g/dL)	32.6 ± 0.8	32.0–36.0
RDW-CV (%)	14.8 (13.8; 16.2)	11.0–15.0
PLT (×10^3^/μL)	193.5 (161.00; 266.50)	125–340
Vitamin D (ng/mL)	21.44 (14.17; 33.36)	30–80
Albumin (g/L)	38 (35; 40.5)	35.0–50.0
Prealbumin (g/L)	0.375 ± 0.112	0.2–0.4
CRP (mg/L)	3.87 (1.8; 12.73)	<5.0
AGP (g/L)	1.345 (1.160; 1.615)	0.5–1.2
Calcium (mmol/L)	2.19 ± 0.22	2.15–2.55
Phosphate (mmol/L)	1.68 ± 0.47	0.81–1.45
iPTH (pg/mL)	316 (119.5; 636.8)	15–65
Sodium (mmol/L)	139.2 ± 2.2	136–145
Potassium (mmol/L)	5.40 ± 0.71	3.5–5.1
Iron (μmol/L)	13.03 ± 4.20	5.83–34.5
TIBC (μmol/L)	39.91 ± 9.35	40.8–76.6
UIBC (μmol/L)	W: 27.06 ± 10.72; M: 29.05 ± 10.09	W: 24.2–70.1; M: 22.3–61.7
sFlt-1 (pg/mL)	129.8 (117.8; 949.1)	63.3–108.3 *

RBC—red blood cells, W—women, M—men, HGB—hemoglobin, HCT—hematocrit, MCV—mean cell volume, MCHC—mean corpuscular hemoglobin concentration, RDW-CV—red blood cell distribution width, PLT—platelets, CRP—C-reactive protein, AGP—α_1_-acid glycoprotein, iPTH—intact parathyroid hormone, TIBC—total iron-binding capacity, UIBC—latent iron-binding capacity, sFlt-1—soluble fms-like tyrosine kinase 1; * range in group of 21 healthy subjects.

**Table 3:** Recommended daily allowance and the evaluation of the studied HD patients’ diet. The values are given as mean ± SD standard deviation or median (Q1; Q3). The diet content was assessed twice, in post-summer and post-winter periods, and the arithmetic mean was calculated from the results.

	RDA	Diet Content in HD Patients (n = 59)	Patients Who Meet RDA, n (%)
Energy (kcal/day)	25–35 kcal/kg ^a^	1400.3 ± 401.3	8 (14)
Total protein (g/day)	1–1.2 g/kg ^a^	52.5 ± 14.9	6 (10)
Animal protein (g/day)	N/A	31.9 ± 10.5	N/A
Plant protein (g/day)	N/A	18.7 (14.5; 22.5)	N/A
Fat (g/day)	20–35% total energy ^b^	49.8 ± 16.8	13 (22) ^c^ 28 (47) ^d^
Carbohydrates (g/day)	45–65% total energy ^b^	189.7 (152.5; 225.9)	10 (17) ^c^ 50 (85) ^d^
Fiber (g/day)	19–65 yo: 25 ^b^ >65 yo: 20 ^b^	13.3 ± 3.9	1 (2)
Calcium (mg/day)	Adjust calcium intake with consideration of use of vitamin D analogs and calcimimetics in order to avoid hypercalcemia or calcium overload ^a^	244.1 (199.0; 341.5)	N/A
Phosphate (mg/day)	Adjust dietary phosphorus intake to maintain serum phosphate levels in the normal range ^a^	748.7 ± 217.9	N/A
Sodium (mg/day)	<2300 ^a^—to reduce blood pressure and improve volume control ^a^. reduced dietary sodium intake—to achieve better volume control and a more desirable body weight ^a^	1513.9 ± 594.6	1. 45 (76) 2. N/A
Potassium (mg/day)	Adjust dietary potassium intake to maintain serum potassium within the normal range ^a^	1976.3 ± 564.7	N/A
Zinc (mg/day)	W: 8 ^b^	5.3 ± 1.7	7 (30)
	M: 11 ^b^	7.6 ± 1.7	0 (0)
Iron (mg/day)	W: 19–50 yo: 18 ^b^ >51 yo: 10 ^b^	6.0 ± 1.8	7 (30)
	M: 10 ^b^	8.0 ± 1.7	1 (3)
Magnesium (mg/day)	W: 19–30 yo: 310 ^b^ >31 yo: 320 ^b^	146.7 ± 47.0	0 (0)
	M: 420 ^b^	189.9 ± 48.8	0 (0)
Copper (mg/day)	0.9 ^b^	0.73 ± 0.22	13 (22)
Manganese (mg/day)	W: 1.8 ^b^	2.8 ± 1.1	20 (87)
	M: 2.3 ^b^	3.2 ± 1.0	25 (69)
Vitamin A (μg/day)	W: 700 ^b^	579.6 ± 261.8	10 (42)
	M: 900 ^b^	621.3 (417.1; 917.8)	5 (14)
β-carotene (μg/day)	Not defined	1393.4 (662.3; 292.4)	N/A
Vitamin B_1_ (Thiamine) (mg/day)	W: 1.1 ^b^	0.7 ± 0.2	6 (26)
	M: 1.3 ^b^	1.0 ± 0.3	6 (17)
Vitamin B_2_ (Riboflavin) (mg/day)	W: 1.1 ^b^	0.7 ± 0.2	6 (26)
	M: 1.3 ^b^	1.03 ± 0.28	4 (11)
Vitamin B_3_ (Niacin) (mg/day)	W: 14 ^b^	9.8 ± 2.9	11 (48)
	M: 16 ^b^	14.84 ± 3.39	9 (25)
Vitamin B_6_ (mg/day)	W: 19–50 yo: 1.3 ^b^ >51 yo: 1.5 ^b^	1.1 ± 0.3	9 (39)
	M: 19–50 yo: 1.3 ^b^ >51 yo: 1.7 ^b^	1.48 ± 0.38	9 (25)
Vitamin B_12_ (µg/day)	2.4 ^b^	1.73 (1.14; 2.74)	16 (27)
Folate (μg/day)	400 ^b^	171 ± 51.7	0 (0)
Vitamin C (mg/day)	W: 75 ^ab^	41.3 (33.2; 68.4)	6 (26)
	M: 90 ^ab^	53.7 (40.2; 88.2)	6 (17)
Vitamin D (µg/day)	15 ^b^	1.6 (0.88; 2.33)	1 (2)
Vitamin E (mg/day)	W: 8 ^b^	5.2 ± 2.4	6 (26)
	M: 10 ^b^	6.9 ± 2.3	1 (3)

^a^ Based on KDOQI 2020 [23]; ^b^ Based on Jarosz M. et al. [24]; ^c^ as calculated from the mean RDA energy intake; ^d^ as calculated from the actual total energy consumption; RDA—Recommended Dietary Allowance, N/A—not available, W—women, M—men, yo—years old.

**Table 4:** Significant correlations between serum concentration of AGP and the results of laboratory tests.

Laboratory Tests	AGP (g/L)	
	R	*p*
CRP (mg/L)	0.66	<0.001
PLT (×10^3^/μL)	0.29	0.028
Iron (μmol/L)	−0.47	<0.001
TIBC (μmol/L)	−0.30	0.023
sFlt-1 (pg/mL)	0.41	0.001

AGP—α_1_-acid glycoprotein, CRP—C-reactive protein, PLT—platelets, TIBC—total iron-binding capacity, sFlt-1—soluble fms-like tyrosine kinase 1.

**Table 5:** Multiple linear regression calculated to assess the variables independently associated with serum AGP (log-transformed serum AGP concentration was the dependent variable). Model 1 (unadjusted) included only the variables significantly associated with log (AGP) in simple analysis, i.e., the variables listed in the Table. Model 2 was additionally adjusted for the following confounders: gender, age, log (dialysis vintage), cardiovascular comorbidity, diabetes, and Kt/V.

Independent Variable	Model 1		Model 2	
	β ± SE	*p*	β ± SE	*p*
log (plant protein intake)	−0.04 ± 0.10	0.6	−0.24 ± 0.14	0.09
log (CRP)	0.50 ± 0.12	<0.001	0.46 ± 0.13	0.001
log (PLT)	0.25 ± 0.10	0.016	0.21 ± 0.11	0.059
Iron	−0.16 ± 0.10	0.1	−0.17 ± 0.11	0.1
log (TIBC)	−0.07 ± 0.11	0.5	−0.13 ± 0.12	0.2
log (sFlt-1)	0.08 ± 0.10	0.4	0.08 ± 0.11	0.5
Regression model	R^2^ = 0.55	<0.001	R^2^ = 0.58	<0.001

β—standardized regression coefficient; SE—standardized error.

## Data Availability

The data are available from the corresponding author upon reasonable request.
